# Therapy of 4T1 breast cancer in mice with Vaccinia virus encoding tumor-associated antigen epitopes and mouse IL2 cytokine

**DOI:** 10.3389/fimmu.2025.1636256

**Published:** 2025-09-18

**Authors:** Mingyu Ye, Ivan Petrov, Ivaylo Gentschev, Eman M. Othman, Aladar A. Szalay

**Affiliations:** ^1^ Cancer Therapy Research Center (CTRC), Department of Biochemistry-I, Biocenter, University of Wuerzburg, Wuerzburg, Germany; ^2^ Department of Radiation Oncology, Rebecca & John Moores Comprehensive Cancer Center, University of California, San Diego, San Diego, CA, United States; ^3^ Department of Pathology, Center of Immune Technologies, Stanford University School of Medicine, San Jose, CA, United States

**Keywords:** cancer immunotherapy, murine mammary carcinoma, engineered vaccinia virus, mIL2, TAA

## Abstract

**Introduction:**

Oncolytic vaccinia virus (VACV) strains are being investigated for use in immunotherapy as a new experimental cancer treatment. Here, we describe the construction, characterization, and use of VACV strains co-expressing murine Interleukin 2 (mIL2) and tumor-associated antigen (TAA)-derived epitopes as potential therapeutic agents against murine mammary carcinoma.

**Methods and results:**

In the 4T1 mouse mammary tumor model, VACV-encoded mIL2 expression remarkably increased CD4+ and antigen-specific CD8+ T cell populations. In addition, the virus-expressed epitopes elicited an antigen-specific T cell response resulting in the inhibition of tumor cell growth. Furthermore, experiments with 4T1 tumor-bearing syngeneic BALB/c mice showed that the mIL2 and TAA-derived epitopes expressing VACV strain achieved a significantly better anti-tumoral response than the VACV strains expressing mIL2 alone.

**Discussion and conclusion:**

Taken together, the combination of concomitant expressions of both compounds is significantly more potent in inhibiting tumor growth than immunotherapy with IL2 alone. These findings suggest that the engineering of novel VACV strains co-expressing IL2 with peptides from tumor-associated antigen epitopes could be a novel strategy for cancer therapy in the future.

## 1 Introduction

VACV is one of the most promising oncolytic viruses for cancer therapy due to its large foreign DNA size carrying capacity (> 25 kb), allowing for the expression of multiple exogenous genes without affecting its natural oncolytic capability (1, 2). Different VACV strains have been extensively used as oncolytic agents for cancer therapy due to their inherent features of tumor tropism, efficient lysis of tumor cells, and ability to spread through tumor tissue, resulting in activation of immune cells (3, 4). Furthermore, VACV is one of the safest viruses, since the viral genome does not integrate into the host cell genome; VACV possesses an excellent safety record historically as a vaccine for eradicating smallpox disease (5). Finally, the use of oncolytic viruses not only leads to the lysis of cancer cells but also stimulates the immune system which attacks the tumor cells (6). Recent studies have confirmed the ability of different oncolytic viruses to increase the anti-tumor immune response (7). These findings have led to an increase in the number of combination immunotherapy approaches (8, 9). In the present study, we developed a novel cancer immunotherapy approach by using an oncolytic VACV co-expressing IL2 with tumor-associated antigen epitopes for the activation and amplification of cytotoxic CD8+ T and CD4+ T cells in mice.

IL2 plays an essential role in the growth and differentiation of immune cells, especially for T cells, B cells and natural killer cells (10). IL2 was the first cytokine in human history used for cancer treatment and it has been approved by the FDA as a monotherapy for renal cell carcinoma and melanoma and was found to mediate tumor regression in human cancer patients (11). The curative effect of IL2 in cancer treatment is dose-dependent, but the main challenge of IL2 in terms of its use as a cancer immunotherapy was that it affects both CD8+ T cells and CD4+Foxp3+ Tregs. This means that IL2 plays a dual role in the immune system both as an immunostimulatory and immunosuppressive agent. Recent clinical studies have indicated that the combination therapy of IL2 with peptide vaccines had increased objective responses in cancer patients compared to IL2 alone, demonstrating that the monotherapy of IL2 can be enhanced by synthetic peptide cancer vaccines (12, 13). One of the important mechanisms for this may be that the administration of IL2 leads to an increase in the numbers of certain tumor antigen-specific T lymphocytes. However, there is very little information available about combination regimens using a Vaccinia virus strain expressing IL2 together with epitopes of tumor-associated antigens (TAAs) for cancer treatment. It has been reported that IL2-expressing Vaccinia virus strains have variable toxicities in mouse models and results in effective anti-tumor responses (14, 15). As opposed to exogenous, synthetic IL2 administration, the population of CD8+ T cells and CD4+ Tregs induced by IL2 expression from the Vaccinia virus has not been extensively studied (16). Here, we investigated the role of mIL2 expressed from an engineered Vaccinia virus Lister strain in 4T1 tumor-bearing syngeneic BALB/c mice and analyzed its effect on CD8+ T cells and CD4+ Tregs.

Peptide-based tumor vaccines are drugs that can elicit tumor antigen-specific immune responses for cancer therapy and the prevention of cancer (17). Tumor cells can be recognized and directly killed by activated CD8+ or CD4+ T cells after the tumor antigens are processed and presented by antigen-presenting cells (APCs) through major histocompatibility complex class I (MHC class I) or class II (MHC class II) molecules (18, 19). TAAs are predominantly self-proteins aberrantly overexpressed in tumors but they are also expressed at lower levels in healthy tissues and in non-cancerous cells (20). Thus, epitopes derived from TAAs may be good sources of peptide vaccines for cancer treatment. It has been demonstrated that TAA peptides expressed by rVACV that minimize antigen processing could elicit therapeutic CTLs *in vivo* (21, 22). Thomson et al. (1995) showed unnatural flanking sequences containing other T cell specific epitopes produced by rVACV did not interfere with the antigen-processing to elicit each corresponding CTL response (23).

Two studies by Franz O. Smith and Steven A. Rosenberg et al. (12, 13) have shown that the combination treatment of IL2 with a TAA epitope vaccine has a significant increase in response rates compared to IL2 alone treatment in patients with melanoma. For example, the combination therapy of a gp100 peptide vaccine plus IL2 significantly improved the overall clinical response compared to the IL2-only group (24). Although simultaneous expression of IL2 and TAA in the same rVACV construct have been studied and indicated that the efficacy of a TAA-produced anti-tumor response can be enhanced by IL2 (25–27), oncolytic rVACVs expressing IL2 and CTL epitopes from TAAs have not yet been comprehensively investigated in tumor therapy. In this report, we constructed novel oncolytic rVACV strains to explore the anti-tumor effectiveness of TAAs-derived peptides and/or IL2 expressed by these virus strains in 4T1 tumor-bearing immunocompetent BALB/c mice. We used several flexible linkers (G4S)2 to connect four peptides derived from two different TAAs to make each epitope spatially equivalent; this structure allows us to introduce more CTL epitopes from different TAAs compared to previous research (24, 26, 27) that only inserted a single tumor antigen with the entire length of the sequence, which may contribute to a more potent anti-tumor response. Additionally, we added an Ig kappa chain leader sequence to the N-terminus of the fusion peptide to facilitate the secretion of the fusion epitopes by virus-infected cells. We found that epitopes of TAAs expressed by oncolytic VACV induced strong peptide-specific T-cell responses aided by IL2. Furthermore, the rVACV expressing both IL2 as well as TAA-derived peptides can significantly improve the therapeutic efficiency against tumors compared to that of an rVACV expressing IL2 alone in tumor-bearing mice.

## 2 Materials and methods

### 2.1 Cell lines and virus

The African green monkey kidney fibroblasts CV-1 (ATCC; CCL-70) and the mouse mammary gland carcinoma 4T1 (ATCC; CRL-2539) cell lines were obtained from the American Type Culture Collection. The mouse mammary carcinoma cell line N2C derived from female BALB-neuT mice was kindly provided by Professor Mario P. Colombo (Fondazione IRCCS Istituto Nazionale dei Tumori, Milano, Lombardia, Italy). The stable cell lines N2C-pTet-turboFP635-EF-1a-Egfp (later called N2C-eGFP) and 4T1-EF-1a-turboFP635 (later called 4T1-turbo) were constructed as described in the Supplementary Data section (**Supplementary Figure S1**). All cell lines were propagated in Dulbecco’s modified Eagle’s medium (DMEM; 11965092, Thermo Fisher Scientific) supplemented with 10% Fetal Bovine Serum (FBS, F4135, Sigma) and 1% penicillin-streptomycin (P4333, Sigma).

### 2.2 Peptides

Peptides utilized in this study; S1:DYIGPCKYI (SPARC_143-151_); S2:MYIFPVHWQF (SPARC_225-234_); AH1-A5: SPSYAYHQF (AH1-A5) were synthesized by Peptide Specialty Laboratories GmbH (Im Neuenheimer Feld 515, 69120 Heidelberg, Germany), at purity levels at least 90% to 95%. The peptide AH1: SPSYVYHQF (gp70_423–431_) was purchased from the company Eurogentec (Catalog: AS-64798, 34801 Campus Drive Fremont, CA 94555, USA), with a purity greater than or equal to 95%. The concentration of stimulating peptide mixture for *in vitro* experiments was 1ug/ml, containing 0.25 ug/ml of each peptide (S1, S2, AH1, and AH1-A5). Sequence information of tumor-associated antigen epitopes are listed in the **Supplementary Table S1**.

### 2.3 Virus strains

Vaccinia virus strain LIVP1.1.1 was derived from LIVP (Lister strain, Institute of Viral Preparations, Moscow, Russia) (28). The L1c-Ig-Turbo was from our laboratory strains collection as previously described (29). Vaccinia virus strain GLV-1h109 virus was derived from the oncolytic vaccinia virus GLV-1h68 by inserting the glaf-1 gene encoding the GLAF-1 antibody under the control of the vaccinia virus promoter (p7.5) into the J2R locus (30). C1-opt1 Vaccinia virus was constructed by insertion of a Turbo-FP635 gene fragment under the control of vaccinia synthetic early/late promoter-Psyn (E/L) into the thymidine kinase (TK) negative Copenhagen (C1) strain, which was provided by Tanja Auth, StemVac GmbH, Bernried, Germany. The construction of the LVP-R-G-mIL2, LVP-R-G-SPARC/gp70-peptides and LVP-R-G-SPARC/gp70-peptides-mIL2 rVACV strains are described in detail in the Results. More details about the strains used in this study can be found in the supplementary files, **Supplementary Figure S2**. All the viruses were expanded in CV-1 cells and purified by sucrose cushion and gradient ultracentrifugation.

### 2.4 Western blot

The Western blot experiments in this work were carried out, as described by Syed R. Haider et al. (31). Samples were separated in 10% acrylamide gels, and the gels were used by semi-dry blotting onto the PVDF membrane (1620177, Bio-Rad). To block the non-specific sites, the membranes were blocked in 5% skim milk powder in 1xTBST (J77500.K2, ThermoFisher) for one hour at room temperature. Then unlabeled primary antibodies were added and incubated overnight at 4°C. After washing three times in 1xTBS 0.5% Triton-X 100 solution, the membranes were incubated with the respective secondary antibodies for one hour at room temperature, followed by scanning in the Bio-Rad ChemiDoc XRS+ system.

### 2.5 Flow cytometry analysis of MHC class I molecule H2-kd and H2-Ld

2x10^5^ (4T1, N2C,4T1-turbo and N2C-eGFP) cells were seeded in four 6-well plates. After three days of incubation, cells were treated with 0.25% trypsin-EDTA (25200056, ThermoFisher) and harvested for the analysis by flow cytometry (Accuri™ C6 Cytometer, BD) using anti-H2-Kd and anti-H2-Ld specific antibodies.

### 2.6 Generation of cell growth curves with real time imaging

2x10^4^ 4T1 and N2C cells were seeded in two individual 24 well plates, and the growth medium was replaced by mIL2 containing medium with different concentrations after 8 hrs. Then a schedule was set to evaluate the cell confluence, in real-time, with the imaging platform-IncuCyte^®^S3 (Essen BioScience, Royston, UK, Cat. no.: 4647). At last, we created the cell proliferation curves through the self-contained software (v2018B) of IncuCyte^®^S3.

### 2.7 Animal experiments

Animal experiments were carried out as described in the protocol approved by the Government of Upper Franconia, Germany and according to the guidelines for the welfare and use of animals in cancer research (application No.: RUF-55.2.2.-2532-2-849). The mice were housed under specific pathogen-free conditions at the Animal Biosafety Level 2 Laboratory of animal facility at University of Wuerzburg (Wuerzburg, Germany). Tumors were generated by implanting 1x10^5^ mammary carcinoma 4T1 cells subcutaneously into the right dorsal flank regions of 5 to 6 weeks-old female BALB/c mice (Charles River, Sulzfeld, Germany). After 13 days, the animals were separated into four groups. The mice were injected intravenously via the tail vein with 1x10^7^ pfu/100µl of LVP-R-G-mIL2 (N = 10 mice), LVP-R-G- SPARC/gp70-peptides-mIL2 (N = 10 mice), C1-opt1 (N = 6 mice) or 100µl PBS (N = 5 mice). After virus treatment, mice were monitored for changes in body weight as well as for signs of toxicity (subcutaneous petechial hemorrhages and lethargy). Mice that developed tumors with volumes close or greater than 1500 mm^3^ were euthanized as required by the animal protocol, and then their splenocytes were analyzed.

### 2.8 Isolation of murine splenocytes

The mice were sacrificed, then spleens were taken out into a 10cm cell culture dish containing 5 ml of ice-cooled complete RPMI medium and crushed using a plunger of the 10-ml syringe until without mostly fibrous tissue remains in the dish. To get the single cell suspension of splenocyte, the cell suspension was pipetted into a 70 μm Nylon cell strainer (352350, Corning) to filter cells and get rid of the tissue debris. After that, the filtered cell suspension was washed with complete RPMI medium by centrifugation. The cells were resuspended in 3 ml of ice-cold ACK buffer (A1049201, ThermoFisher) to lyse the red blood cells and the sample was incubated at room temperature for 5 min with occasional shaking. The lysis reaction was stopped by adding 7 ml complete RPMI medium. Lastly, the cells were washed with complete RPMI medium twice and resuspended in 5 ml of complete RPMI medium. The samples were maintained in ice for the remainder of the experiment.

### 2.9 Flow cytometry analysis of splenocytes

The pelleted splenocytes were resuspended in the Cell Staining Buffer (420201, BioLegend) at 5-10x10^6^ cells/ml and distributed 100µl/well of cells (5 – 10 x 10^5^ cells/tube) into a 96 well plate. For Fc receptor blocking, 100µl/well Anti-Mouse CD16/CD32 antibody (10µg/ml) was added to the plate after removing the cell staining buffer. After blocking the non-specific sites,100µl/well of appropriate pairs of conjugated antibodies (CD3+CD4+; CD3+CD8+; CD4+PD-1+; CD8+PD-1+) and corresponding isotypes were added to the plate. After 15 – 20 min. of incubation, the cell pellets were resuspended in 0.2 ml of Cell Staining Buffer after being washed twice to perform the flow cytometric analysis.

### 2.10 Isolation of CD8+ T cells

After the preparation of single splenocyte pellet suspensions from the spleen of PBS or rVACV injected mice, the cells were filtered through a 70µm cell strainer (352350, Corning) and washed once with the MojoSort™ Buffer (480135, BioLegend). To deplete the non CD8+ T cells, a biotin antibody cocktail was added by incubation with magnetic Streptavidin Nanobeads (480135, BioLegend). Then the magnetically labelled fraction was retained by a magnetic separator, and the CD8+ T cells were collected by decanting the liquid in a clean tube.

### 2.11 Detection of IFN-γ by flow cytometry

CD8+ T cells isolated from spleens were seeded into a 96-well U-bottom cell culture plate (4 x 10^5^ per well). Then 200 μl per well of prepared stimulation medium (Complete RPMI medium with 1ug/ml peptide mixture, 160 U/ml mouse IL2 and 6 ug/ml anti-mouse CD28 antibody) and negative control medium (Complete RPMI medium with 160 U/ml mouse IL2 and 6 ug/ml anti-mouse CD28 antibody) were added to the plate for IFN-γ induction. After 8 – 72 hrs of incubation, the IFN-γ was blocked in the cytoplasm by adding Brefeldin A to perform the flow cytometric analysis.

### 2.12 Activation and expansion of epitope specific memory CD8+ T cells

First, we prepared CD8+ T cell activation medium. Negative control medium: Complete RPMI medium with 160 U/ml IL2 and 6 ug/ml anti-mouse CD28 antibody. Activation medium: Complete RPMI medium with 1ug/ml peptide mixture,160 U/ml IL2 and 6 ug/ml anti-mouse CD28 antibody. Secondly, an aliquot of 1 x 105 cells/ml 50μL isolated CD8+ T cells was placed per well into a U-bottom 96-well plate, then 150μL of activation medium was added. Lastly, the plate was placed into the CO2 incubator and the cells were cultured for 3 – 4 days to active and expand epitope specific memory CD8+ T cells (effector T cells).

### 2.13 Co-cultivation assay of activated CD8+ T cells with target tumor cells

After determining the cell count, 1 x 10^3^ per well target cells (4T1-EF-1a-turboFP635 and N2CpTet- turboFP635-EF-1a-Egfp stable cell line) with complete RPMI medium were added to a flat bottom 96-well plate for overnight incubation. Then 4-5x10^3^ effector T cells (i.e., epitope pre-activated or inactivated CD8+ T cells) with fresh activation medium or negative control media were added to the plate containing the target cells. The plates were put into the IncuCyte^®^S3 and a scan schedule was set up that scanned the plate once every 4 hrs with the Red or Green fluorescent signal. Lastly, the data were analyzed with Incucyte^®^S3 Software (v2018B).

### 2.14 Antibodies used in flow cytometry and western blot

All antibodies used in flow cytometry and Western blot in this study are listed in the **Supplementary Table S2**.

### 2.15 Statistical analyses

All the data analyses and graphics were performed using Graphpad Prism 8.0 software. Results are presented as mean ± SD (*, P < 0.05; **, P < 0.01; ***, P < 0.001; ****, P< 0.0001, ns: not significant). The Shapiro-Wilk normality test was performed for all datasets to analyze whether each dataset followed a normal distribution pattern. If the dataset followed a normal distribution, we applied parametric tests such as the student’s t-test and one-way analysis of variance. The student’s t-test was used for non-paired comparisons of two groups. Differences between more than two groups were determined by one-way analysis of variance (ANOVA), and the Kaplan-Meier method with the log-rank test used for survival analysis. A p-value <0.05 was considered statistically significant.

## 3 Results

### 3.1 Design and construction of recombinant Vaccinia virus strains expressing tumor-associated antigen-derived peptides and/or mIL2

**Figure 1:**
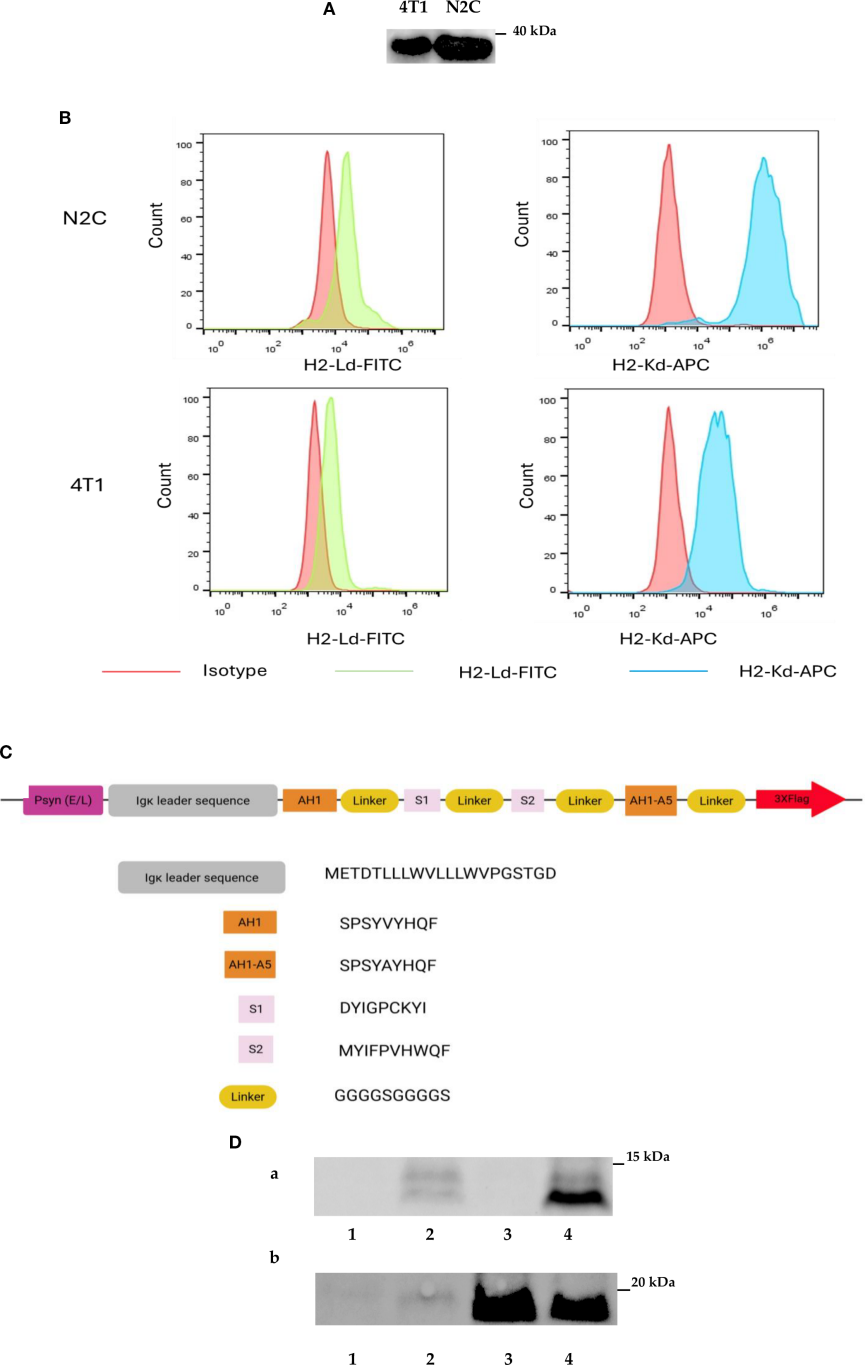
Design and construction of recombinant Vaccinia viruses. **(A)** Expression of SPARC in 4T1 and N2C mammary cancer cell lines detected by Western blotting. **(B)** Detection of H2-Kd and H2-Ld expression by FACS analysis in 4T1 and N2C cell lines. **(C)** Schematic representation of the design of the tumor-associated fusion protein SPARC/gp70. **(D)** Detection of SPARC/gp70-fusion protein and mIL2 expression in different strains of rVACVs infected 4T1 cells via Western blot analyses. **(A)** SPARC/gp70-fusion protein detection with anti-Flag antibody. **(B)** IL2 detection with mouse anti-IL2 antibody. Line 1: 4T1 cells infected with C1-opt1; Line 2: 4T1 cells infected with LVP-R-G-SPARC/gp70-peptides; Line 3: 4T1 cells infected with LVP-R-G-mIL2; Line 4: 4T1 cells infected with LVP-R-G-SPARC/gp70-peptides-mIL2.

The 4T1 cell line is a mouse mammary carcinoma cell line that is widely used in triple-negative breast cancer research as a model for human breast cancer. Our previous findings showed that the cancer therapeutic function of IL2-expressing VACV in the 4T1 mouse model depends not only on the backbone virus strain (Lister strain 1.1.1 vs Copenhagen strain) but also on the promoter used to drive the expression of the payload (p7.5 promoter vs Psyn (E/L) promoter) (**Supplementary Figure S3**). These data indicate that the abundance of IL2 expressed from VACV may be important for the success of tumor treatment. To investigate the combination therapy of IL2 with a peptide vaccine expressed by the same VACV, we used VACV Lister strain 1.1.1 as the backbone to generate a new strain of rVACV with the mouse IL2 gene expression driven by Psyn (E/L) promoter, and one strain of IL2 expressing virus with the cloned tumor antigen epitope DNA. Peptide-based vaccines are used in breast cancer therapy, and more than a dozen of breast cancer antigens have been well defined (32). In our study, we chose the SPARC protein (33) and the Murine leukemia virus (MuLV) gp70 protein (34) as the tumor-associated antigen epitopes sources for cancer treatment in the mouse mammary cancer model. The S1 and S2 peptides are H2-Kd-restricted epitopes from tumor-associated antigen SPARC, stimulating anti-tumor immunity without causing autoimmune disease in mouse mammary tumor models (35). The H2-Ld-restricted epitope AH1 was derived from the MuLV gp70 protein expressed in many murine tumor cell lines, including the 4T1 cell line (34, 36, 37). AH1-A5 peptide was derived from AH1 peptide, in which Ala in position 5 of AH1 was replaced by Val and even had a more robust immune response than the AH1 peptide (38). Therefore, before constructing the TAAs-derived peptides expressing rVACV, we evaluated the expression of the SPARC protein and MHC class I molecules H2-Kd and H2-Ld in mammary cancer cell lines 4T1 and N2C by western blot analysis and flow cytometry. The data demonstrated clearly that the SPARC protein as well the MHC class I molecule H2-Kd and H2-Ld were expressed in both tested cell lines (**Figures 1A, B**). In the next step, we designed a new fusion protein consisting of an N-terminal secretion signal (Ig kappa chain leader sequence), four different tumor-associated antigen epitopes (AH1, S1, S2 and AH1-A5) connected by four flexible linker (G4S)2 domains and three C-terminal FLAG tag elements allowing easy detection of the protein (**Figure 1C**, **Supplementary Tables S3A, B**). The DNA sequence encoding this SPARC/gp70 peptides fusion protein under the control of the Psyn (E/L) of VACV was inserted into the site between locus 157 and locus 158 of the LIVP1.1.1 genome to generate LVP-R-SPARC/gp70-peptides expressing strain. Finally, a fragment of the mIL2 gene under the control of the Psyn (E/L) promoter was inserted into the TK locus to generate the LVP-R-G SPARC/gp70-peptides-mIL2 strain (**Supplementary Figure S2**). To construct LVP-R-G-mIL2 strain, we introduced the mIL2 gene driven by the Psyn (E/L) promoter into the TK locus of the L1c-Ig-Turbo virus. The expression of mIL2 and SPARC/gp70 epitope fusion protein was verified by the Western blot (**Figure 1D**) and Elisa (**Supplementary Figure S4**) analysis in the viruses infected 4T1 cells. Additionally, Paciotti and Tamarkin reported that IL2 does directly inhibit the growth of human breast cancer cell lines *in vitro* and *in vivo* (39). Therefore, we used the IncuCyte^®^S3 system for real-time monitoring of the proliferation of 4T1 and N2C cells after treatment with different concentrations of mIL2 in cell culture. The results showed that increasing concentrations of mIL2 do inhibit both 4T1 and N2C cell growth under these cell culture conditions (**Supplementary Figures S5A, B**).

### 3.2 Therapy of 4T1 tumor-bearing mice with oncolytic rVACVs expressing tumor antigen epitopes and/or mIL2

**Figure 2:**
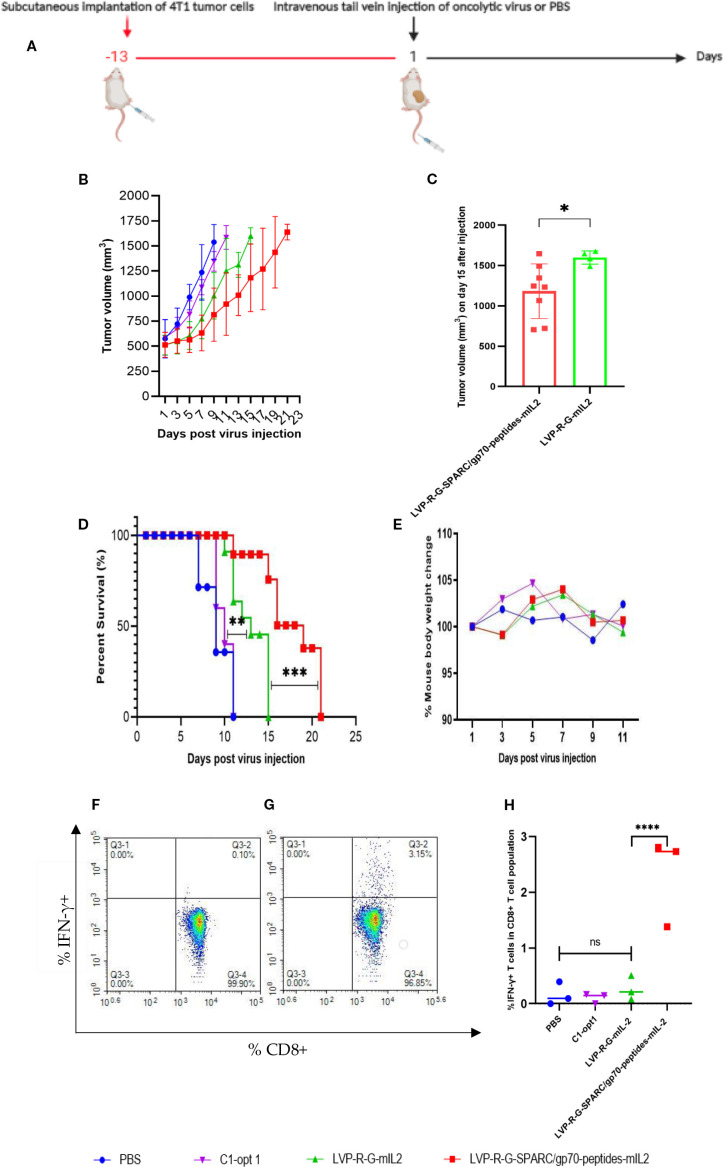
Effects of rVACVs treatment on 4T1 tumor-bearing BALB/c mice after intravenous application of different VACV constructions. **(A)** Schedule of tumor cells implantation and rVACV administration for tumor therapy in 4T1 tumor-bearing BALB/c mice. **(B)** Tumor growth of 4T1 tumor-bearing BALB/c mice after treatment with rVACVs or PBS. **(C)** Comparison of tumor volume of LVP-R-G-SPARC/gp70-peptides-mIL2 or LVP-R-G-mIL2 injected mice at the day 15. The statistical significance was calculated by the t-test, * indicates P<0.05. **(D)** Survival of 4T1 tumor-bearing BALB/c mice after VACVs or PBS treatment. The comparison of survival time between the four different treated groups was statistically evaluated by Kaplan-Meier and log-rank (Mantel-Cox) tests (Graphpad Prism, San Diego, CA). P<0.05 was considered statistically significant. ** indicates P<0.01, *** indicates P<0.001, **** indicates p<0.0001. **(E)** Effect of different treatments on the weights of 4T1 tumor-bearing BALB/c mice. **(F-H)** FACS analysis of IFN-γ produced CD8+ T lymphocytes isolated from rVACVs injected mice. **(F)** IFN-γ detection of CD8 T lymphocytes isolated from LVP-R-G-mIL2 injected mice and induced by peptide mixture. **(G)** IFN-γ detection of CD8 T lymphocytes isolated from LVP-R-G-SPARC/gp70-peptides-mIL2 injected mice and induced by peptide mixture. **(H)** Comparison of IFN-γ expression by CD8+ T lymphocytes of different group mice after rVACV or PBS treatment. The statistical significance was calculated by ordinary two-way ANOVA test, * indicates P<0.05 was considered statistically significant. ** indicates P<0.01, p values > 0.05 are not significantly different (ns).

**Figure 3:**
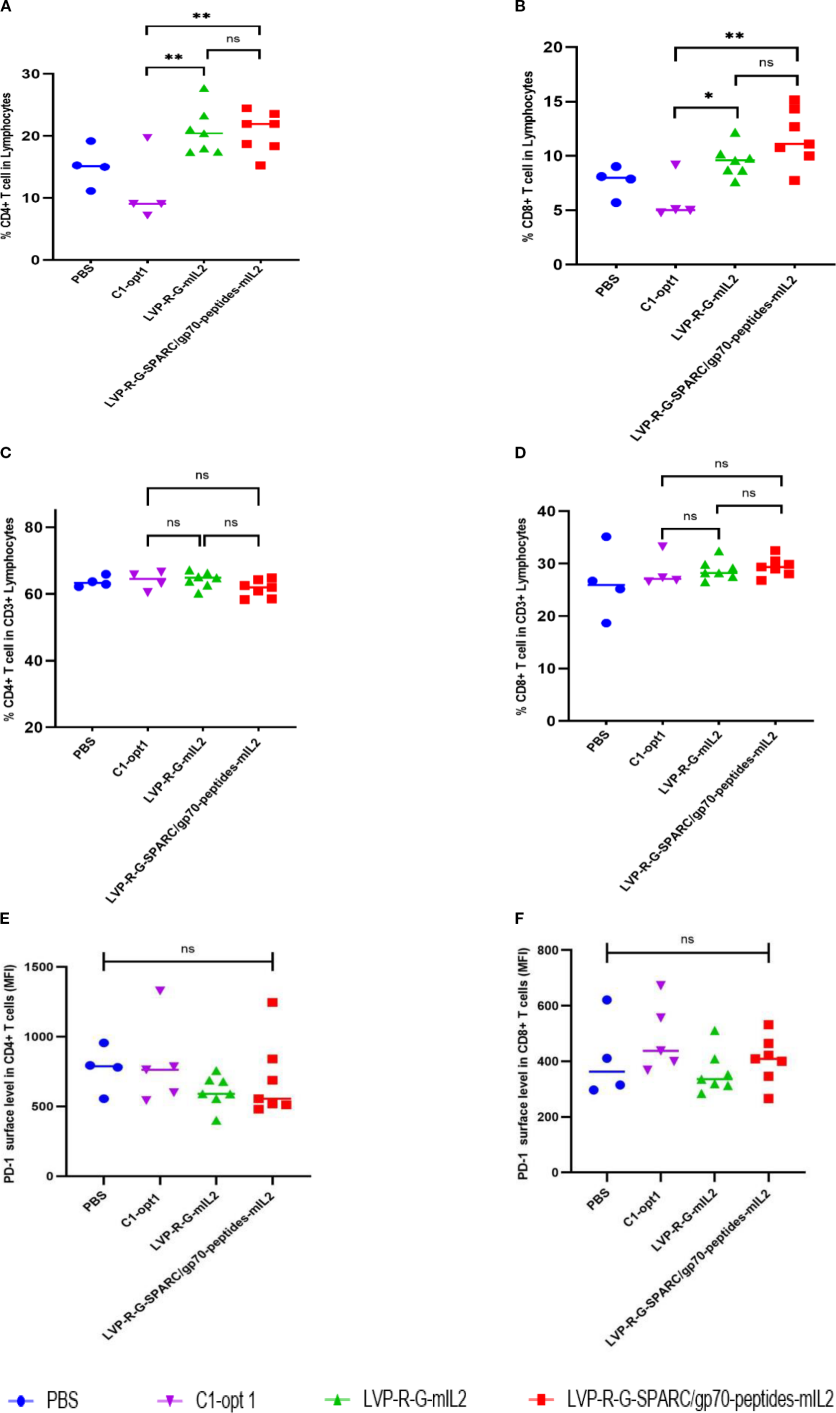
Investigation of CD4+/CD3+ and CD8+/CD3+ T cells populations in the lymphocyte fraction. **(A-F)** FACS analysis of splenocyte preparations from mice after injection with PBS or rVACV Strains. The statistical significance was calculated by ordinary one-way ANOVA test, * indicates P<0.05 was considered statistically significant. ** indicates P<0.01, p values > 0.05 are not significantly different (ns). **(A)** Percent [%] of CD4+cells in splenic lymphocyte populations. **(B)** Percent [%] of CD8+ cells in lymphocytes population. **(C)** Percent [%] of CD4+cells CD3+ lymphocytes population. **(D)** Percent [%] of CD8+cells CD3+ lymphocytes population. **(E)** PD-1 expression as mean fluorescence intensity (MFI) on CD4+ T cells. **(F)** PD-1 expression as mean fluorescence intensity (MFI) on CD8+ T cells.

For the evaluation of the therapeutic potential of the newly constructed rVACVs, a 4T1 tumor-bearing syngeneic BALB/c mouse model was established. For this purpose, 1 x 105 mammary carcinoma 4T1 cells were implanted into the right dorsal flank of 5- to 6-week-old female BALB/c mice. After 13 days, the animals were separated into four groups. The mice were injected intravenously into the tail vein with 1×10^7^ pfu/100µl of LVP-R-G-mIL2 (n=10), LVP-R-G-SPARC/gp70-peptides-mIL2 (n=10), C1-opt1 (n=10; virus control group) or with 100µl PBS (n=5; negative control group) (**Figure 2A**). In order to evaluate the toxicity and further characterize anti-tumor effects of the different rVACVs, the mouse body weight and tumor volumes were measured and monitored every three days until the day of termination. Mice were euthanized when the tumors reached a volume of about 1500 mm3. Data from animal experiments proved that the VACV strain expressing mIL2 plus tumor-associated antigen epitopes show a more efficient anti-tumor response than the VACV strain expressing mIL2 alone. The LVP-R-G-SPARC/gp70-peptides-mIL2 strain may significantly inhibit tumor growth compared to the LVP-R-G-mIL2 strain (**Figures 2B–D**). In these experimental settings, the expression of mIL2 driven by the Psyn (E/L) promoter in VACV Lister strain had minimal toxicity in tumor-bearing mice, according to the evaluation of mouse body weight change (**Figure 2E**). To assess the antigen-specific CD8+ T cells, we analyzed IFN-γ expression through flow cytometry to quantify the ratio of gp70 protein- and SPARC-specific CD8+ cells in the T lymphocyte fraction. The result shows that a fraction of CD8+ cells from peptide over-expressing vaccinia virus injected mice secrete IFN-γ after specific peptide mixture (see peptides in materials) stimulation. In contrast, CD8+ T cells from the other groups did not produce abundant IFN-γ (**Figures 2F–H**). These findings demonstrated that the structure of the SPARC/gp70 fusion protein preserves the function of the T cell epitope. To investigate cell populations of CD4+ and CD8+ T cells in the lymphocyte fraction, isolated splenocytes from different strains of rVACV-vaccinated mice were analyzed by FACS. The results showed that both CD4+ and CD8+ cell populations were significantly expanded in the mIL2 treated mouse groups (**Figures 3A, B**), while the ratio of CD4+/CD3+ and CD8+/CD3+ did not show a significant change (**Figure 3C, D**). In parallel, to assess whether VACV-induced stimulation can rescue the exhausted T cells, we also investigated the expression of PD-1 in both CD4+/CD3+ and CD8+/CD3+ T cell populations. The results indicate no significant differences in PD-1 expression levels on T cells (**Figures 3E, F**). Collectively, this animal experiment demonstrated that the mIL2 expression driven by the Psyn (E/L) promoter (LVP-R-G-SPARC/gp70-peptides-mIL2 or LVP-R-G-mIL2 injected mice) remarkably increased both CD4+ and CD8+ T cell populations in vivo, but did not affect the expression of PD-1 molecule on CD4+ or CD8+ T cells.

### 3.3 Assessment of specific anti-tumor CD8+ T cell cytotoxicity through co-culture assays

**Figure 4:**
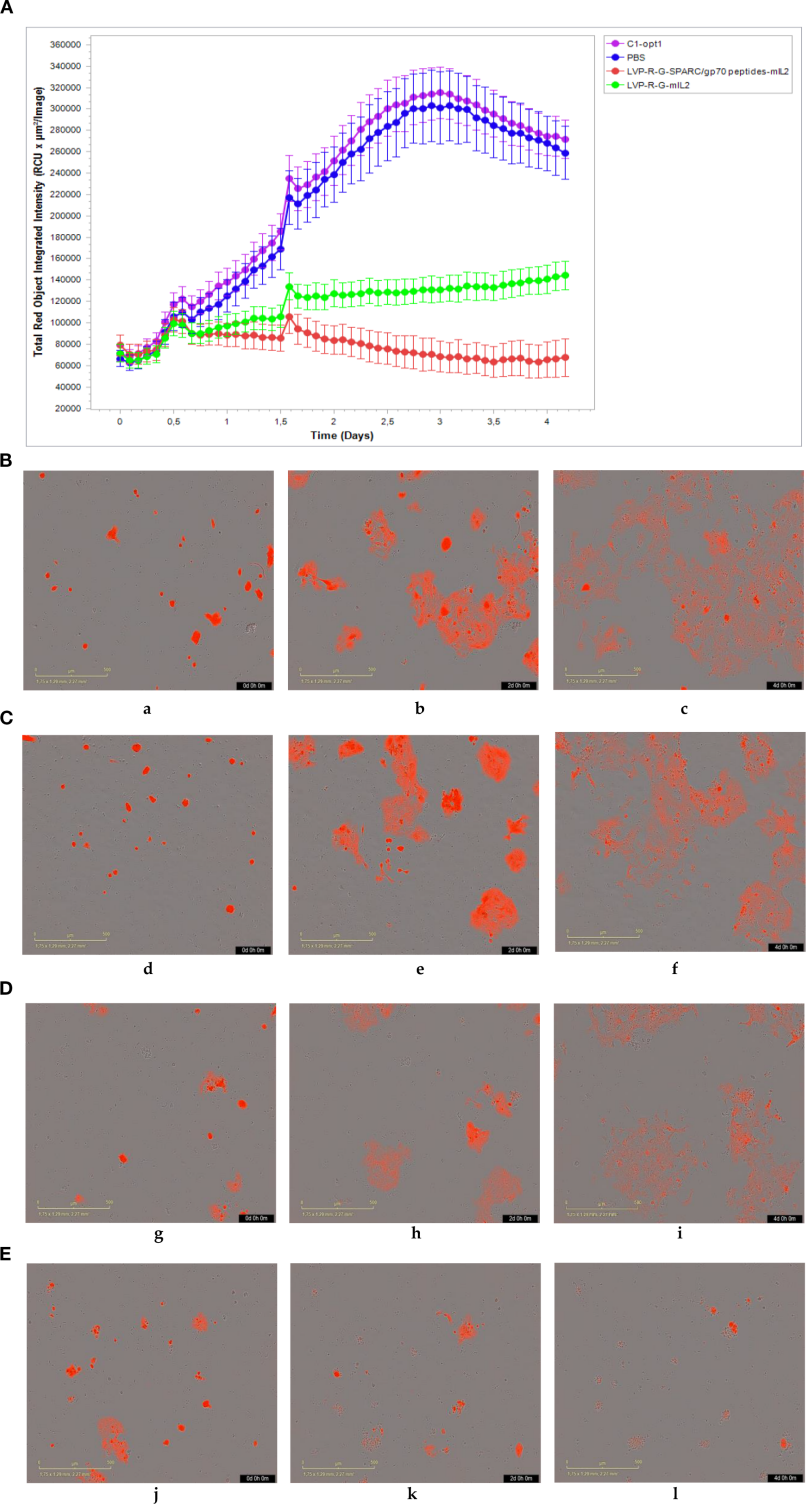
Effect of cytotoxic CD8+ T cells on the 4T1-turbo. CD8+ T cells were isolated and purified from different groups of mice, co-cultured with target cells, and then scanned by IncuCyte^®^S3 at different time points.**(A-E)** CD8+ T cells co-cultured with 4T1-turbo cells in the medium containing peptide mixture and anti-CD28 antibody. **(A)** The cell proliferation curve of 4T1-turbo cells co-cultured with the CD8+ T cells. **(B)** CD8+ T cells purified from PBS group (a-0d,b-2d,b-4d). **(C)** CD8+ T cells purified from C1-opt1 group (d-0d,e-2d,f-4d). **(D)** CD8+ T cells purified from LVP-R-G-mIL2 group (g-0d,h-2d,i-4d). **(E)** CD8+ T cells purified from LVP-R-G-SPARC/gp70 peptides-mIL2 group (j-0d,k-2d,l-4d).

**Figure 5:**
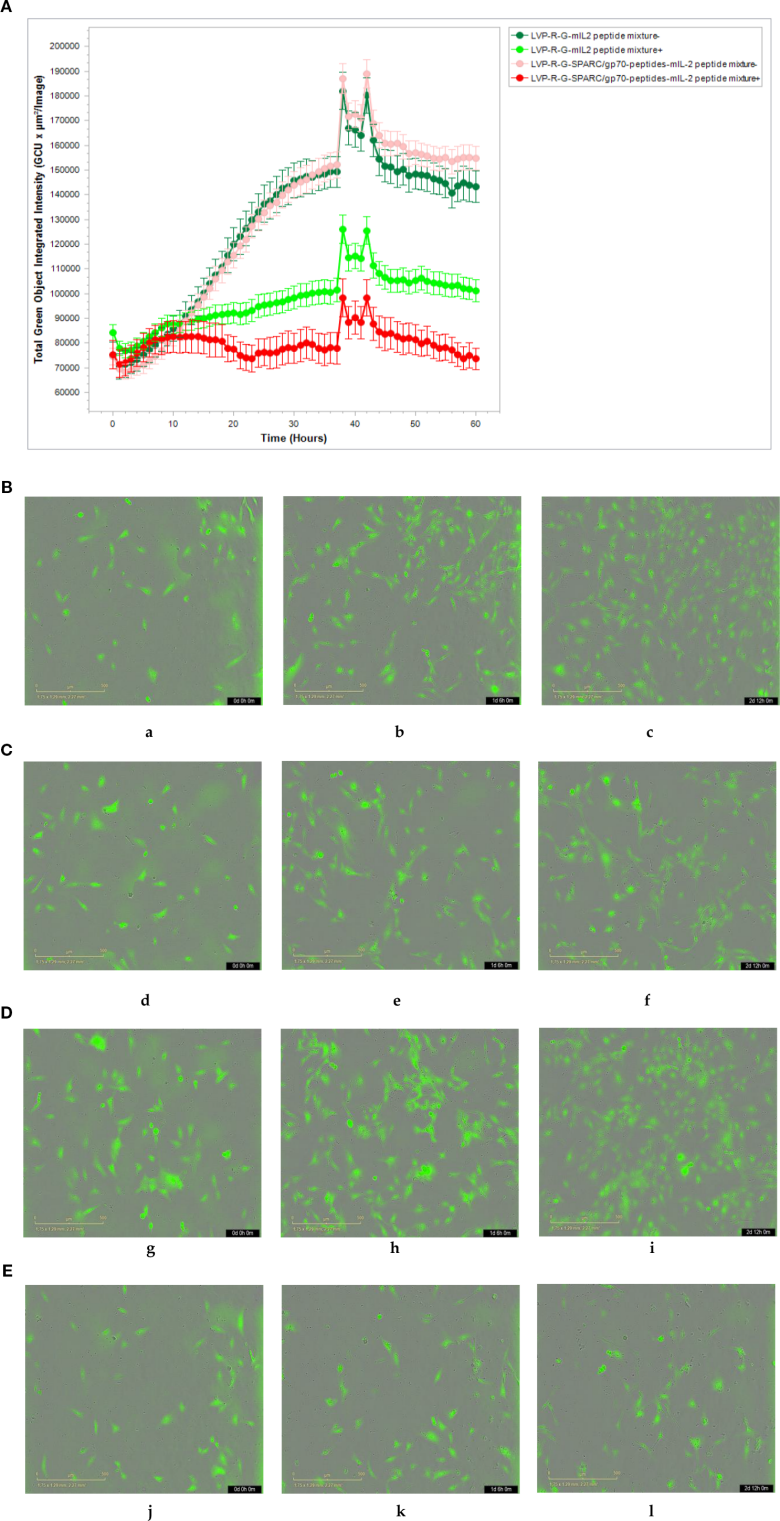
Effect of cytotoxic CD8+ T cells on the N2C-eGFP cell lines. **(A-E)** CD8+ T cells co-cultured with N2C-eGFP cells in medium either containing or not containing peptide mixture and anti-CD28 antibody.**(A)** The cell proliferation curve of N2C-eGFP cells co-culture with the CD8+ T cells. **(B)** CD8+ T cells purified from LVP-R-G-mIL2 group without peptide mixture inducement (a-0h,b-30h,b-60h). **(C)** CD8+ T cells purified from LVP-R-G-mIL2 group with peptide mixture inducement (d-0h,e-30h,f-60h). **(D)** CD8+ T cells purified from LVP-R-G-SPARC/gp70 peptides-mIL2 group without peptide mixture inducement (g-0h,h-30h,i-60h). **(E)** CD8+ T cells purified from LVP-R-G-SPARC/gp70 peptides-mIL2 group with peptide mixture inducement (j-0h,k-30h,l-60h).

**Figure 6:**
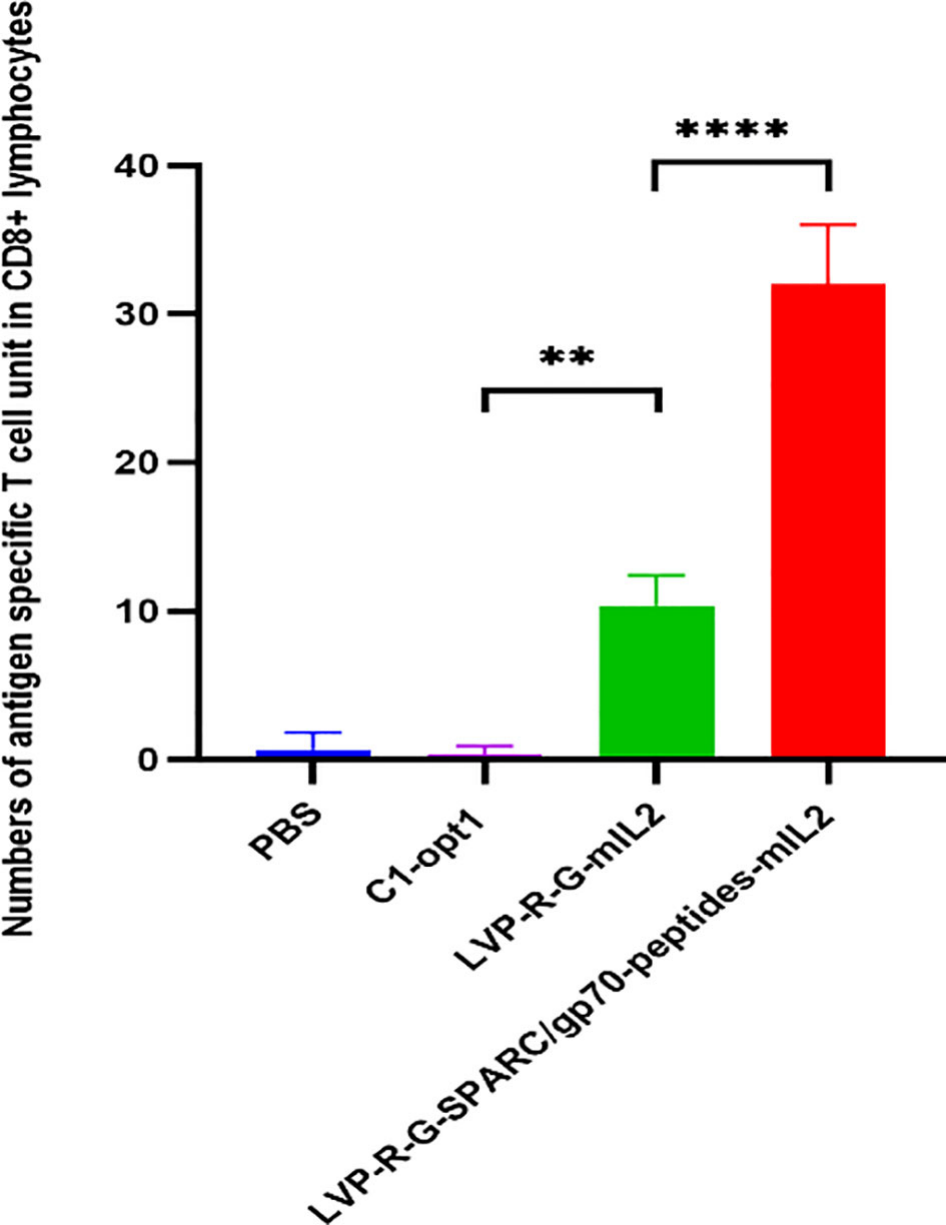
Effect of VACV expressed IL2 on antigen-specific cytotoxic T cells. Number of antigen-specific cytotoxic T cell units in CD8+ lymphocytes. The statistical significance was calculated by ordinary one-way ANOVA test, * indicates P<0.05 was considered statistically significant. ** indicates P<0.01, p values > 0.05 are not significantly different (ns).

CD8+ T cell-mediated cytotoxicity plays a critical role in the anti-tumor immune responses of the peptide-based vaccines. Therefore, ex vivo testing of interactions of tumor cells and T cells is pivotal for evaluating the effectiveness of the cancer vaccines (40). To assess antigen-specific CD8+ T cell cytotoxicity, we developed a co-culture assay to visualize the cell-killing using a real-time imaging platform-IncuCyte^®^S3. In this experimental setting, CD8+ T cells were isolated and purified from the spleens of rVACVs and PBS treated mice. Then these pre-activated, effector cells were co-cultured with the target tumor cells (4T1-turbo and N2C-eGFP) in the context of a peptide mixture and anti-CD28 antibody in 96-well plates to induce antigen-specific lytic responses. These target cells are two modified mammary cancer cell lines expressing fluorescent proteins that help to indicate the T-cell induced tumor cell lysis. For videos, images, and specific lysis curves generation, the plates were put into IncuCyte^®^S3 to monitor the effector-to-target cell interaction at the different time points. After activation and expansion of epitope specific memory CD8+ T cells (effector cells) through peptide mixture and anti-CD28 antibody, we first detected the effects of cytotoxic CD8+ T cells on the 4T1-turbo cell line by using an optimal ratio of target cancer cells to effector cells (4-5:1). The results show that CD8+ T cells isolated from both mIL2 injected mouse groups induced the lysis of target cells, especially in the peptide mixture plus mIL2 combination group which had close to 100% killing efficiency. In contrast, the PBS and C1-opt1 groups did not limit the growth of cancer cells (**Figures 4A-E**, **Supplementary Video 1**). Secondly, we investigated the cytotoxic function of CD8+ T cells in eGFP-expressing N2C cells with or without the peptide mixture inducement in the content of anti-CD28 antibody. The data show that CD8+ T cells isolated from peptide expressing rVACVs injected mice did not kill the N2C-eGFP cells without peptide mixture mediation, but significantly inhibited cell growth after treatment with peptide mixture. The killing efficiency was significantly higher than that of target cells cultured with CD8+ T cells which were isolated from non-peptide-expressing rVACVs injected mice (**Figures 5A–E**, **Supplementary Video 2**). In addition, to compare the amount of VACV expressed IL2 stimulated antigen-specific CD8+ T cell population in vivo with the non-exogenous IL2 stimulated intrinsic T cells, we consider a 4T1-turbo cell cluster being lysed as a unit of activated antigen-specific cytotoxic CD8+ T cells in the images of the co-culture assays that were post cultured between 2 and 12 hours (**Supplementary Figure S6**). The quantity of antigen-specific CD8+ T cells was then assessed by counting the number of these units in various animal groups. The results reveal that the number of active CD8+ T cells in the IL2-only expression group is higher than in the no IL2 expression group. As expected, the cytotoxic CD8+ T cells were sparse in the PBS group, which was similar to the C1-opt1 group. The peptides plus IL2 group was significantly greater than the other constructs (**Figure 6**).

## 4 Discussion

The therapeutic benefits of IL2 regarding cancer are often associated with severe toxicities, supporting Tregs were reported to play critical roles in suppressing the immune response (41–43). Even though IL2 monotherapy was not as successful as expected in improving patient survival, the combination therapy of IL2 and other anti-cancer agents showed encouraging clinical results, especially in combination with peptide cancer vaccines. In the combination treatment setting, the low intrinsic immunogenicity of the individual epitopes was strongly enhanced by IL2 administration. The mechanism for this was uncovered by Hussein Sultan et al. recently (44). Here, we utilized the vaccinia virus as an oncolytic vector to express tumor-associated peptides and/or mIL2 cytokine to treat murine mammary cancer.

IL2 is the first effective cytokine used in human cancer immunotherapy. The mechanism of systemic IL2 therapy in cancer immunotherapy includes the stimulation of lymphoid proliferation in tissues and the activation of host-derived T cells *in vivo* (45). Several studies indicate that systemic administration of IL-2 *in vivo* has anticancer effectiveness (46, 47). Vaccinia virus has been investigated as a vector for the transfer of immunotherapeutic genes (cytokines, antibodies, etc.) (48–50). Systemic administration of VACV expressed IL-2 is one of the most studied cytokines. However, the effect of intravenous injected VACV expressed IL-2 on splenocytes T cells was not extensively investigated. The curative effect of IL2 in cancer treatment is dose-dependent, and HD IL2 was always necessary to achieve clinical efficacy. Our previous data suggested that the mIL2 expression driven by a pSyn (E/L) promoter in the TK locus of VACV Lister strain did lead to tumor reduction in the 4T1 mouse tumor models. Thus, we have chosen the VACV Lister strain as the backbone of recombinant virus construction, and mIL2 transcription is initiated by the Psyn (E/L) promoter in the TK locus. Our data presented here demonstrates that a mIL2-expressing rVACV can help in expanding both CD4+ and CD8+ T cell populations in mice spleen, although it did not change the ratio of CD4+/CD3+ and CD8+/CD3+ cells. These findings may indicate that mIL2 expressing rVACV has even greater anti-tumor effectiveness than the control groups. Additionally, we investigated mean fluorescence intensity (MFI) of PD-1 expression of CD4+ and CD8+ cell populations in all four animal groups and did not find significant differences among these groups. This suggests that whereas IL-2 and tumor-associated peptides produced by VACV can activate CD8+ T cells via cytokine support and TCR interaction, they cannot rescue the exhausted T cells.

In this study, we selected four T cell epitopes derived from the SPARC protein and the MuLV gp70 protein as TAA-derived peptides to be constitutively expressed by VACV in order to elicit a peptide-specific cytotoxic T lymphocytes response. All four peptides are potential cancer vaccine candidates (35–37). Umer et al. recently showed that even minor changes to the VACV genome may impact the immune reaction in a way that affects the efficiency of tumor therapy (51). Therefore, to avoid the alteration of the immune response, based on our former model, we inserted DNA fragment encoding the four T cell epitopes into a VACV “no expression” intergenic region between the intergenic locus 157 and intergenic locus 158. All the foreign proteins of interest expressed by the engineered rVACV were identified via Western blots and/or fluorescent microscopy. The CTL epitopes expressed by the rVACV successfully induced a number of IFN-γ-producing CD8+ lymphocytes, confirmed by ex vivo stimulation with the chemically synthesized peptide mixture. Therefore, based on these findings, we can predict that the fusion peptide expressed by VACV is able to induce the activity of cytotoxic CD8+ effector T cells, according to the ex vivo data presented above, and therefore the structure of the fusion protein provides a new strategy for the tumor antigen-based peptide vaccine design.

Potentiation of the tumor-specific killing ability of CD8+ T cells *in vivo* is one of the most critical points for successful immunotherapies. Tumor antigen epitopes encoding rVACV has been demonstrated that could elicit robust antitumor CD8+ T cells without the necessity of *in vitro* sensitization (22, 23, 52). However, there was no visible evidence demonstrated that the epitopes induced CD8+ T cells can directly lysis the target tumor cells in the rVACV expression system. Here, we developed a novel co-culture assay that can be easily applied by using a real-time imaging platform-IncuCyte^®^S3 to monitor the process of cell death induced by the antigen-specific CD8+ T cells. Using this method, we isolated CD8+ T splenocytes from rVACV vaccinated mice and tested the cytotoxic activity of CD8+ T cells against 4T1-turbo and N2C-eGFP cell lines under the stimulation of tumor antigens. As expected, the mIL2 plus peptide mixture stimulation increased the numbers of tumor antigen-specific CD8+ T cells and potently induced the death of target tumor cells. Furthermore, we found that the rVACV expressing tumor-associated peptides can greatly enhance the therapeutic effects of mIL2, in combination drastically inhibiting the growth of the tumor. In addition, we have proven that the tumor antigen-specific T cells efficiency was epitope-dependent. According to the comparison of numbers of antigen-specific CD8+ T cells from the C1-opt1 and LVP-R-G-mIL2 vaccinated mouse groups, we found that mIL2 expressed from rVACV significantly increased the number of antigen-specific CD8+ T cells. Taken together, the combination of VACV-mediated tumor therapy with CTL epitopes and IL2 may be a very promising, novel choice for enhancing the cytotoxic T cell response against tumors.

The oncolytic vaccine virus is relatively safe, as has been proved by hundreds of experimental data in preclinical and clinical studies. Although we have demonstrated the tumor therapeutic efficacy of VACV strains co-expressing IL2 with tumor-associated antigen epitopes in a mouse model—offering a promising strategy for oncolytic cancer immunotherapy—the translation of this concept into human clinical trials remains challenging. In this study, we utilized T cell epitopes derived from TAAs; however, the overexpression of TAAs may lead to the risk of autoimmunity, as these antigens are also expressed on normal healthy tissues. Additionally, T cells capable of recognizing self-antigens may be eliminated due to immune tolerance mechanisms in the host. Therefore, personalized tumor-specific antigens (TSAs) could represent a superior alternative for translating this strategy into human cancer therapy, as they are uniquely expressed on tumor cells, potentially reducing the risk of autoimmunity while enhancing antitumor immune responses. Moreover, although we did not observe significant IL-2-associated toxicity in our mouse studies, IL2 toxicity remains an important consideration for clinical application. Notably, modified forms of IL2 have been reported to reduce toxicity while increasing its half-life *in vivo*, thereby enhancing its ability to target specific immune cell populations (53, 54). Thus, using novel engineered IL-2 variants instead of the wild-type version could be a promising approach to reduce systemic toxicity while enhancing the therapeutic efficacy of VACV-based oncolytic immunotherapy in the clinic.

## Data Availability

The original contributions presented in the study are included in the article/**Supplementary Material**. Further inquiries can be directed to the corresponding authors.
