# Supplementary material for: Therapy of 4T1 breast cancer in mice with Vaccinia virus encoding tumor-associated antigen epitopes and mouse IL2 cytokine
Source: Front Immunol. 2025 Sep 18;16:1636256. doi: 10.3389/fimmu.2025.1636256 (PMC12488624; doi:10.3389/fimmu.2025.1636256)
Supplement: Supplementary file 1 [file DataSheet1.pdf]

Supplementary Material

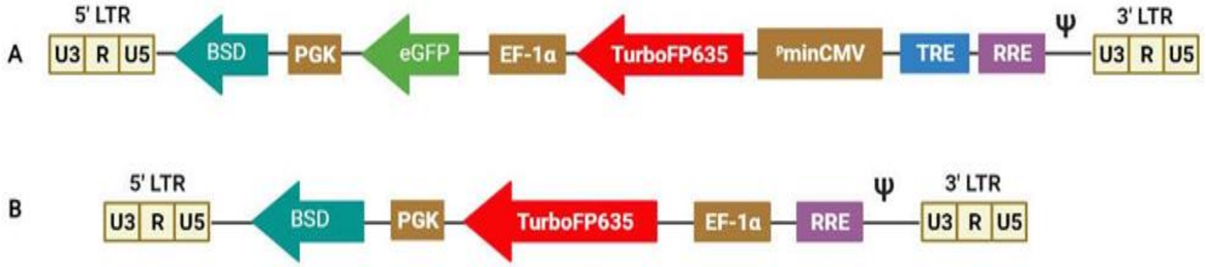

Figure S1. Schematic presentation of stable cell lines constructed by lentiviral vector. (A) N2C- pTet-turboFP635-EF-1a-Egfp cell line and (B) 4T1-EF-1a-turboFP635 cell line. PGK: Phosphoglycerate kinase promoter. BSD: Blasticidin. pminCMV: minimal CMV promoter. EF-1α: EF-1 alpha promoter. RRE: Rev response elements. TRE: Transcriptional response elements.

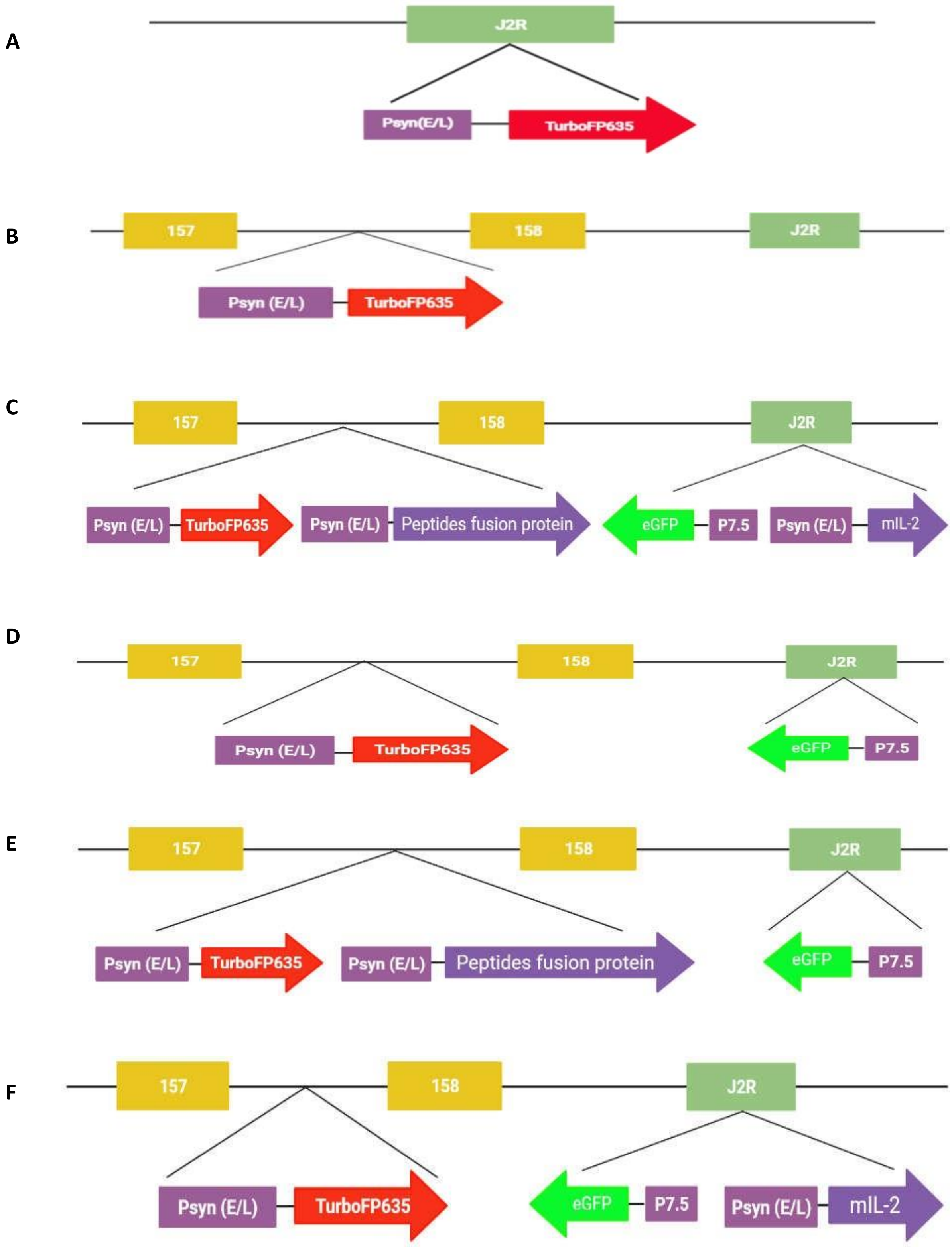

Figure S2. Genetic map of rVACV strains. (A): C1-opt1. (B): L1c-Ig-Turbo. (C): L1c-Ig-Turbo-SPARC/gp70 peptides-TK-Egfp-mIL2(LVP-R-G-SPARC/gp70 peptides-mIL2). (D): L1c-Ig-Turbo-TK-Egfp (LVP-R-G). (E): L1c-Ig-Turbo-SPARC/gp70 peptides-TK-eGFP (LVP-R-G-SPARC/gp70 peptides). (F): L1c-Ig-Turbo-TK-Egfp-mIL2(LVP-R-G-mIL2). J2R: J2R locus of Vaccinia virus genome, Encoding Thymidine Kinase. 158: locus of Vaccinia virus

genome. 157: locus of Vaccinia virus genome. Psyn(E/L): synthetic early/late promoter. P7.5: VACV promoter p7.5.

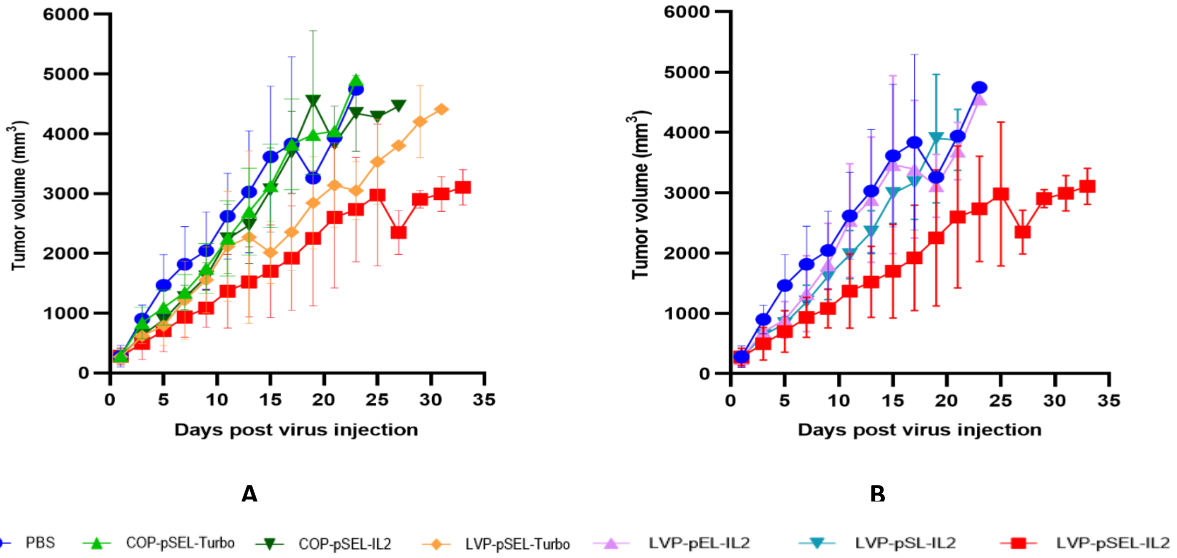

Figure S3. Therapeutic efficacy of VACV expressing mIL2 on 4T1 tumor-bearing BALB/c mice through IV application. (**A**) Lister 1.1.1 strains vs Copenhagen 15.1.1 strains. (**B**) Lister 1.1.1 strains expressing mIL2 under different promotes. COP: Copenhagen strain backbone viruses; LVP: Lister strain backbone viruses; pEL: P7.5 early/late promoter; pSL: P7.5 later promoter; pSEL: Synthetic early/late promoter; Turbo: TurboFP635; mIL2: mouse interleukin 2. BALB-c mice were cared for and maintained according to animal care regulations under Genelux approved protocol by the Institutional Animal Care and Use Committee of Explora Bio labs, Inc (San Diego, CA, USA; protocol number: EB14-09B).

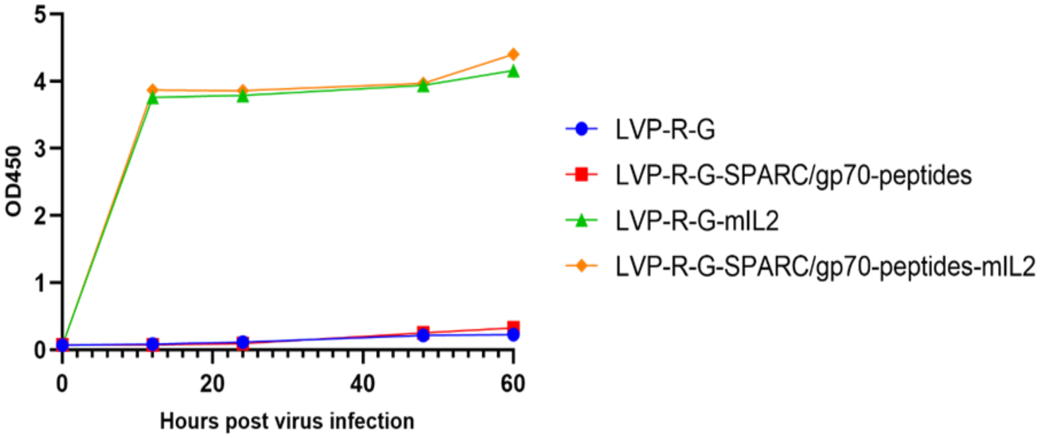

Figure S4. Detection of mIL2 expression in different strains of rVACVs infected 4T1 cells via Elisa analyses. The 4T1 cells were infected by different rVACV strains at an MOI of 0.5, and after 2–3 days of infection, then the supernatant was collected and analysed by a mouse IL-2 Elisa kit.

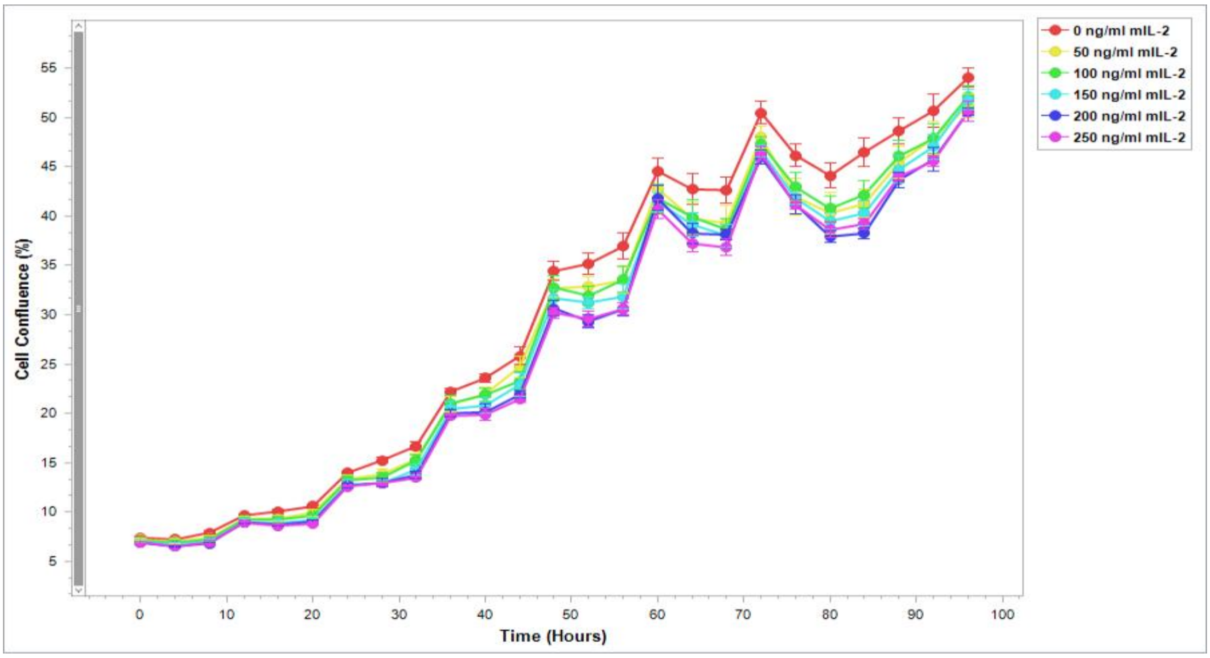

A

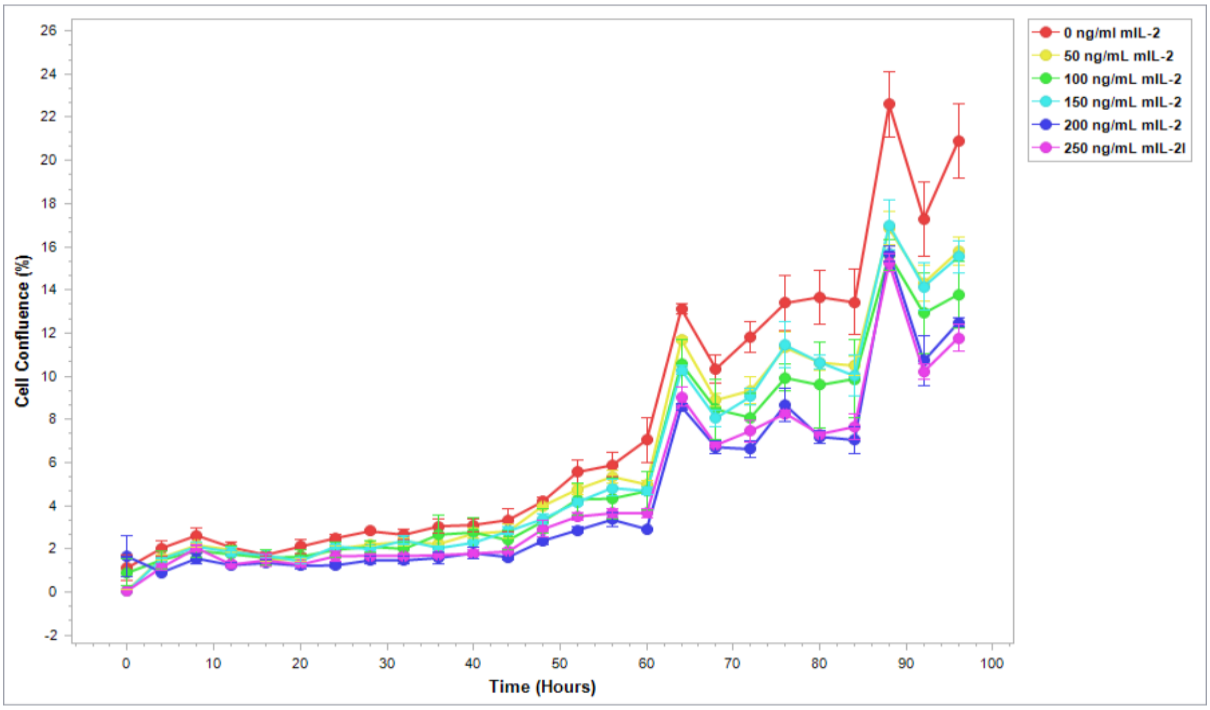

B

Figure S5. Proliferation of mIL2 treated cancer cells. 4T1 cells (**A**) or N2C cells (**B**) were cultured with the medium containing different concentration of mIL2 (0ng/ml, 50ng/ml, 100ng/ml, 150ng/ml, 200ng/ml, and 250ng/ml) for four days, continuously.

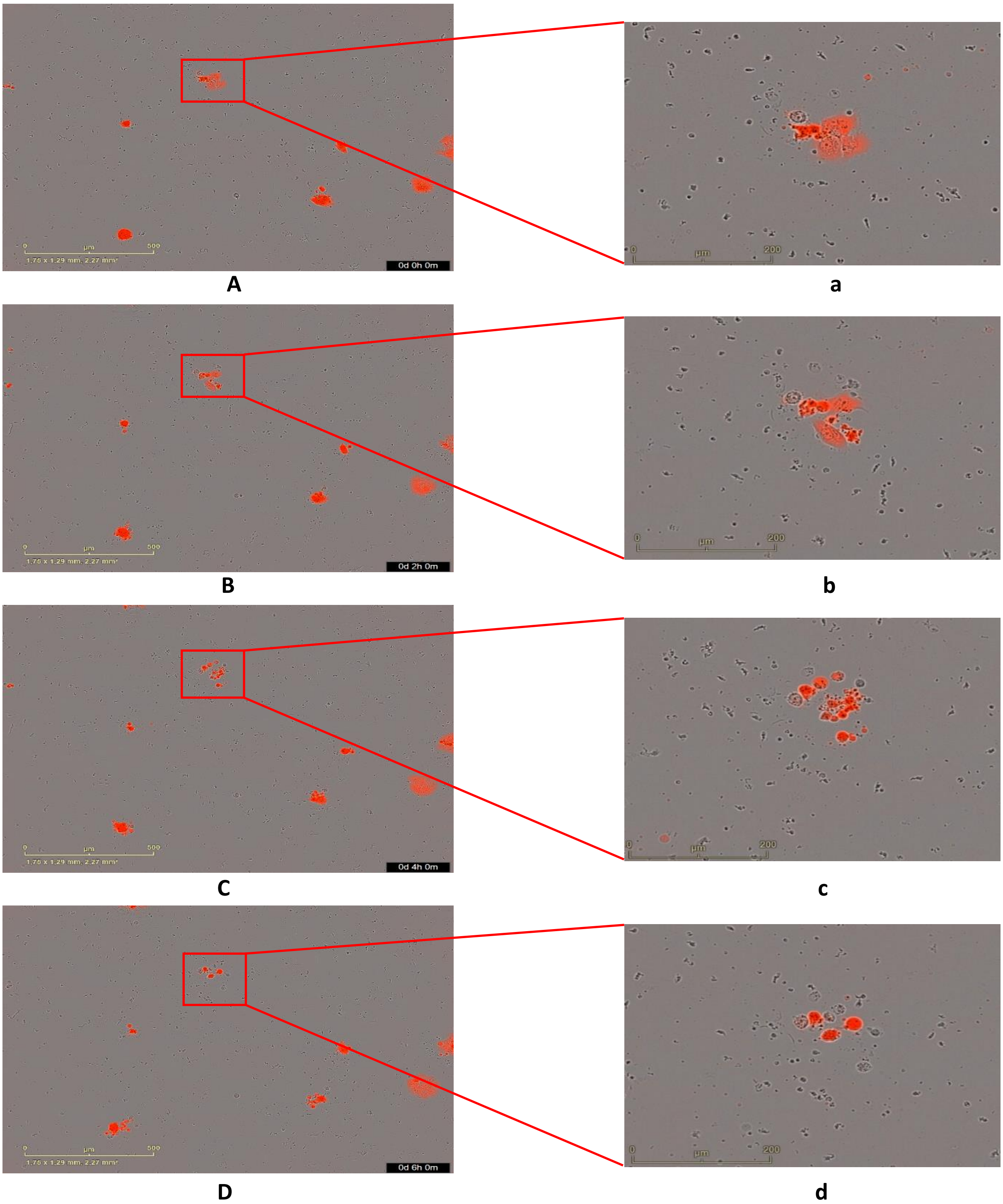

Figure S6. Unit of antigen-specific cytotoxic T cells in CD8+ lymphocytes, which show in the red squares. (**A**) CD8+ T cells purified from LVP-R-G-SPARC/gp70 peptides-mIL2 group co-culture with the 4T1-turbo cells in the inducement of peptides mixture and anti-CD28 antibody at 0-hour point. (**a**) Image enlargement (IE) from (**A**). (**B**) At the 2-hour point. (**b**) IE from (**B**). (**C**) At the 4-hour point. (**c**) IE from (**C**). (**D**) At the 6-hour point. (**d**) IE from (**D**).

Table S1. Sequence information of tumor-associated antigen epitopes

Short name	Source of epitopes	Position	Peptide sequence	Mouse Haplotype	Mouse Strains	TAA	T cell Specificity
S1	SPARC	143–151	DYIGPCKYI	H2-Kd	BALB/c	Yes	CD8+
S2	SPARC	225–234	MYIFPVHWQF	H2-Kd	BALB/c	Yes	CD8+
AH1	MuLV gp70	423-431	SPSYVVYHQF	H2-Ld	BALB/c	Yes	CD8+
AH1-A5	MuLV gp70	423-431	SPSYAYHQF	H2-Ld	BALB/c	Yes	CD8+

Table S2. Antibodies used in flow cytometry and Western blot analysis

**Name of antibody**	**Manufacturer**	**Catalogue number**
APC anti-mouse CD279 (PD-1) Antibody	BioLegend	135210
APC Rat IgG2a, κ Isotype Ctrl Antibody	BioLegend	400511
APC anti-mouse IFN-γ R β chain Antibody	BioLegend	113605
APC anti-mouse CD3ε Antibody	BioLegend	100312
APC Armenian Hamster IgG Isotype Ctrl Antibody	BioLegend	400911
Alexa Fluor® 488 anti-mouse CD8a Antibody	BioLegend	100723
Alexa Fluor® 488 Rat IgG2a, κ Isotype Ctrl Antibody	BioLegend	400525
FITC anti-mouse CD4 Antibody	BioLegend	100406
FITC Rat IgG2b, κ Isotype Ctrl Antibody	BioLegend	400605
FITC Mouse IgG2a, κ Isotype Ctrl Antibody	BioLegend	400207
Purified anti-mouse CD28 Antibody	BioLegend	102102
Purified Rat Anti-Mouse CD16/CD32 (Mouse BD Fc Block™) Clone 2.4G2 (RUO)	BD	553141
MHC Class I (H-2Kd) Monoclonal Antibody (SF1-1.1.1), APC, eBioscience™	Thermo Fisher	17-5957-82
APC Mouse IgG2a, κ Isotype Ctrl	BioLegend	981906
H-2Ld Monoclonal Antibody (30-5-7S), FITC	Thermo Fisher	MA5-18006
FITC Mouse IgG2a, κ Isotype Ctrl	BioLegend	981902
Anti-Flag M2 antibody	Sigma-Aldrich	F3165
DylightTM 649 Goat anti-mouse IgG	BioLegend	405312
IL-2 Monoclonal Antibody (5H4)	Thermo Fisher	MM101
IL-2 Mouse Uncoated ELISA Kit with Plates	Thermo Fisher	88-7024-22
APC Goat anti-rat IgG (minimal x-reactivity) Antibody	BioLegend	405407
Anti-SPARC antibody	R&D	AF942-SP
Rabbit Anti-Goat IgG H&L (HRP)	Abcam	ab6741
Cell Staining Buffer	BioLegend	420201
Fixation Buffer	BioLegend	420801
Intracellular Staining Permeabilization Wash Buffer	BioLegend	421002
Brefeldin A Solution (1,000X)	BioLegend	423301

Table S3. Sequence of elements in the fusion protein

Name of elements	Sequence
Ig Kappa leader sequence	METDTLLLWVLLLWVPGSTGD
$(G_4S)_2$ Linker	GGGGSGGGGS
3XFlag	DYKDHDGDYKDHDIDYKDDDDK
